# Time-Course of Changes in Choroidal Thickness after Complete Mydriasis Induced by Compound Tropicamide in Children

**DOI:** 10.1371/journal.pone.0162468

**Published:** 2016-09-13

**Authors:** Zhouyue Li, Junwen Zeng, Wei Jin, Wen Long, Weizhong Lan, Xiao Yang

**Affiliations:** 1 State Key Laboratory of Ophthalmology, Zhongshan Ophthalmic Center, Sun Yat-sen University, Guangzhou, China; 2 Optometry and Visual Science Institute, Aier Eye Hospital Group, Changsha, Hunan Province, China; University of Zürich, SWITZERLAND

## Abstract

**Purpose:**

The aim of this study was to investigate the time-course of changes in choroidal thickness (ChT) following complete mydriasis induced by compound tropicamide.

**Methods:**

ChT was measured by OCT with the enhanced-depth imaging technique (Spectralis HRA+OCT, Heidelberg Engineering, Germany) at nine locations of the fundus: subfoveal ChT (SFChT) and ChT at 1 mm and 3 mm from the fovea in four quadrants. Mydriasis was induced with compound tropicamide (0.5% tropicamide plus 0.5% phenylephrine hydrochloride, three doses at 5-minute intervals). Measurements were conducted prior to the instillation and at 0, 30, and 60 min following complete mydriasis. Results at different time-points were compared using repeated-measures ANOVA to investigate the time-course of the changes.

**Results:**

Thirty-nine subjects (mean age 11.9±2 years; 16 males and 23 females) were enrolled in the study. Compound tropicamide resulted in a statistically significant decrease in SFChT at 0, 30, and 60 min after complete mydriasis, as compared to baseline (−5±4 μm, −12±4 μm, and −13±4 μm, respectively; all P<0.0001). No significant changes were detected in the parafoveal choroid except at 1 mm temporal (T_1mm_) and nasal (N_1mm_) to the fovea at 30 and 60 min (T_1mm_: −6±4 μm and −7±5 μm at 30 and 60 min; N_1mm_: −6±4 μm and −7±5 μm at 30 and 60 min, respectively; all P<0.0001). Repeated-measures ANOVA showed a significant interaction between the time after complete mydriasis and the effect of the mydriasis agent.

**Conclusions:**

Complete mydriasis induced by compound tropicamide led to choroidal thinning, and the magnitude varied over time.

## Introduction

As a highly vascularized layer located between the retinal pigment epithelium (RPE) and the sclera, the choroid plays an important role in normal ocular function. With the highest rate of blood flow in any structure of the human body, the choroid not only supplies oxygen and nourishment to the outer retina, but also secretes growth factors [1]. In addition, the choroid has been shown to directly modulate the intraocular pressure (IOP) by providing an extra pathway for the drainage of aqueous fluid from the anterior chamber (the uveoscleral pathway) [2].

With the emergence of optical coherence tomography (OCT), the choroid is no longer a blind area to detect. With OCT, researchers have found subtle changes in choroidal thickness (ChT) to be associated with a number of ocular diseases, including central serous chorioretinopathy [3], high-myopia-related chorioretinal atrophy [4], age-related macular degeneration [5], and polypoidal choroidal vasculopathy [6]. ChT imaging *in vivo* has become an essential step in monitoring diseases related to the choroid. Additionally, new advances in OCT, such as 1060-nm OCT and enhanced-depth imaging (EDI) techniques, have enabled researchers to detect deeper and clearer images of the ocular fundus [7–9]. For instance, with EDI-OCT, it was reported that in subjects older than 60 years of age, subfoveal ChT (SFChT) becomes significantly thinner with increased age, and the degeneration speed was calculated as precisely as 5.40 μm per year [10]. In addition to the association with a number of ocular diseases as mentioned above, ChT is likely to play an important role in emmetropization—the choroid can adjust the position of the retina to meet the focal point of the imposed retinal defocus, by changing its thickness [11–13]. Thus, ChT has recently been attracting increased attention [14–16].

However, ChT in humans is found to be affected by multiple factors. Besides the imposed retinal defocus, factors such as age [17], refractive error [8,18], diurnal fluctuation [19–21], visual stimulus (e.g., imposed retinal blur [11–13]), and even smoking [22] and caffeine intake [23] have been reported to influence ChT. Topical administration of adrenergic and anticholinergic agents, which are usually used to induce mydriasis and/or cycloplegia in clinical practice, were recently found to have an impact on ChT [24–27]. The compound tropicamide eye drop, consisting of 0.5% tropicamide mixed with 0.5% phenylephrine hydrochloride, is one of the most common agents used in China prior to fundus examinations or for refraction in children. In this study, we investigated whether and to what extent ChT would change with the administration of this common mydriatic agent.

## Material and Methods

### Subjects

Thirty-nine healthy children (16 males and 23 females) with a mean age of 11.9±2 years were enrolled in this study. The children were participants in an ongoing clinical trial (Register No. ChiCTR-IPR-14005505, www.chictr.org.cn/showproj.aspx?proj=9873) evaluating the efficacy and safety of a novel orthokeratology lens. Prior to the clinical trial, written consent was obtained from all children and their parents after a thorough explanation of the purposes and risks of all procedures throughout the clinical trial, including the current experiment. The study was conducted in accordance with the tenets of the Declaration of Helsinki and was approved by the ethics committee of Zhongshan Ophthalmic Center, Sun Yat-sen University (permit number: 2015QXNL003).

Before the study, each participant underwent an ophthalmic examination to ensure ocular health. All subjects had normal visual acuity of logMAR 0.00 or better. The mean spherical equivalent refractive error was −2.82±0.68 DS (range: −2.63~−4.25 DS). To avoid the potential confounding influence of ocular diurnal variations [20], all procedures were performed at approximately the same time of day (between 3 pm and 6 pm) throughout the study, in a quiet and dim environment.

### Procedures

#### Preparation for examination

Mydriasis was achieved by instilling one drop of compound tropicamide in each eye (0.5% tropicamide plus 0.5% phenylephrine hydrochloride; Xing Qi Ophthalmic Co., Ltd., Shenyang, China), in three doses at 5-min intervals. Direct and indirect light reflexes were detected 30 min after the last eye drop administration. No light reflex was confirmed before the following examinations were performed. Otherwise, an additional drop of the agent was administered until the light reflex was absent.

#### Measurement I

**Fig 1:**
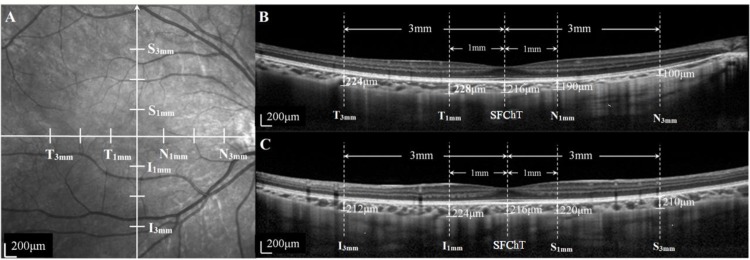
Illustration of choroidal thickness (ChT) measurements in the fundus. (A) Fundus image showing horizontal and vertical scan lines going through the fovea. Choroidal thicknesses at 1 and 3 mm to the fovea superiorly, inferiorly, temporally, and nasally are marked as S_1mm_, I_1mm_, T_1mm_, N_1mm_, S_3mm_, I_3mm_, T_3mm_, and N_3mm_, respectively. (B) OCT image with horizontal scan. Left: temporal; right: nasal. OCT image shows SFChT, T_1mm_, N_1mm_, T_3mm_, and N_3mm_. (C) OCT image with vertical scan. Left: inferior; right: superior. OCT image shows SFChT, S_1mm_, I_1mm_, S_3mm_, and I_3mm_. Short horizontal lines in each dash-line in (B) and (C) indicate the borders measured for the choroid, and each figure indicates individual ChT in different locations.

Prior to the administration of the eye drops (pre-treatment) and 0, 30, and 60 min after complete mydriasis, choroidal scans were obtained of each patient’s right eye using an EDI combined spectral-domain OCT (Spectralis HRA+OCT, Heidelberg Engineering, Heidelberg, Germany; wavelength for scan: 870 nm). This instrument has a lateral resolution of 6 μm and an axial resolution of 5 μm. The EDI image was averaged over 100 scans using the automatic averaging and eye-tracking system. This instrument has an Auto Rescan feature, which tracks features on the instrument’s scanning laser ophthalmoscope (SLO) retinal image in order to ensure that follow-up scans are done at the same retinal location as the baseline measurements. ChT was measured by a masked and experienced investigator, with the border of ChT defined as extending from the outer portion of the hyperreflective line (corresponding to the RPE) to the inner surface of the sclera. ChT was measured at nine different locations on the retina (Fig 1): at the fovea and at 1 mm and 3 mm from the fovea in the nasal (N_1mm_ and N_3mm_, respectively), temporal (T_1mm_ and T_3mm_, respectively), superior (S_1mm_ and S_3mm_, respectively), and inferior (I_1mm_ and I_3mm_, respectively) quadrants.

#### Measurement II

In addition to ChT as measured by OCT, a non-contact biometer (Lenstar LS 900; Haag Streit AG, Koeniz, Switzerland) was also applied to measure other ocular biometrics, including axial length (AL), anterior chamber depth (ACD), lens thickness (LT), and corneal curvature (K). AL, as defined by the instrument, refers to the distance between the anterior cornea and the RPE.

These two measurements were conducted only in the participants’ right eyes. To avoid the potential sequence bias on the results, the order of the two measurements for each participant was randomly arranged at each time-point. To ensure the precision of every parameter, each measurement was conducted twice.

### Data analysis

All statistical analyses were performed using SPSS version 16.0 (SPSS 16.0, Inc., Chicago, IL, USA). All data were reported as averages ± standard deviations (SD), unless otherwise stated. The change of the parameter refers to the difference between the baseline value and the value detected at a certain time-point after complete mydriasis. A repeated-measures analysis of variance (ANOVA) was performed to investigate the time-course of the changes in the abovementioned parameters. P<0.05 at two tails was considered to be statistically significant. The test-retest SD collected at each session was calculated in accordance with the method of Sander [24]. The interclass correlation coefficient (ICC), an index of measurement reliability, was also calculated for each of the ocular parameters. In agreement with the suggestion by Portney and Watkins [28], ICC>0.75 was regarded as excellent measurement reliability, ICC≥0.4 as good reliability, and ICC<0.4 as poor reliability.

## Results

### Test-retest repeatability of measurements

The average test-retest variability for each ocular variable between two subsequent series is illustrated in Table 1. Highly precise measurements were generally indicated by the mean difference of the test-retest results for all variables. The test-retest SD in this study ranged from 3.55 to 3.80 μm for SFChT and parafoveal ChT, 0.01 to 0.02 mm for ocular biometrics (AL, ACD, and LT), and 0.07 D to 0.12 D for K values. The ICC analysis suggested excellent reliability for all variables (all ICC >0.90).

**Table 1:** Summary of test-retest repeatability for ocular variables.

	Mean difference of test-retest	SD of test-retest	95% limit of agreement	ICC
AL (mm)	0.00	0.01	[−0.03,0.03]	0.999
ACD (mm)	0.00	0.01	[−0.02,0.03]	0.998
LT (mm)	0.00	0.02	[−0.03,0.03]	0.993
K_1_ (D)	0.00	0.07	[−0.14,0.14]	0.998
K_2_ (D)	0.02	0.12	[−0.22,0.25]	0.994
SFChT (μm)	3.90	3.55	[−3.05,10.85]	0.998
Parafoveal ChT at 1.0 mm (μm)	4.73	3.69	[−2.50,11.95]	0.998
Parafoveal ChT at 3.0 mm (μm)	4.98	3.80	[−2.47,12.43]	0.999

Each measure was conducted twice. SD: standard deviation. ICC: interclass correlation coefficient.

### Time-course of changes of ChT after complete mydriasis

Table 2 shows the changes in ChT at different locations of the retina at different time-points after complete mydriasis. It was shown that complete mydriasis induced significantly thinner SFChT, by −5±4 μm compared with baseline (paired t-test, P<0.001). In addition, this change became significantly obvious with observation time (repeated-measures ANOVA, P<0.001), then stabilized after 30 min (−12±4 μm at 30 min vs −13±4 μm at 60 min; paired t-test, P = 0.144).

**Table 2:** Effects of compound tropicamide on ChT after complete mydriasis.

Location	Mean change from baseline			ANOVA
	0 min	30 min	60 min	
	Mean±SD (P *)			P ^#^
SFChT (μm)	−5±4 **(0.000)**	−12±4 **(0.000)**	−13±4 **(0.000)**	**0.000**
Temporal 1 mm (μm)	−1±6 (0.359)	−6±4 **(0.000)**	−7±5 **(0.000)**	**0.000**
Superior 1 mm (μm)	−2±4 **(0.002)**	−6±4 **(0.000)**	−7±4 **(0.000)**	**0.021**
Nasal 1 mm (μm)	−2±4 **(0.002)**	−6±4 **(0.000)**	−7±4 **(0.000)**	**0.000**
Inferior 1 mm (μm)	−1±5 (0.153)	−3±6 **(0.000)**	−4±5 **(0.000)**	**0.000**
Temporal 3 mm (μm)	0±4 (0.488)	−2±5 **(0.000)**	−2±5 **(0.000)**	**0.034**
Superior 3 mm (μm)	0±6 (0.913)	−1±6 (0.363)	−2±5 **(0.007)**	0.271
Nasal 3 mm (μm)	0±3 (0.437)	−1±3 **(0.002)**	2±3 **(0.000)**	**0.049**
Inferior 3 mm (μm)	0±5 (0.707)	−1±5 (0.144)	−2±4 **(0.000)**	0.293

The time-points were 0, 30, and 60 min after complete mydriasis. Positive values represent increased ChT, while negative values correspond to decreased ChT.

* indicates the significance of the change from baseline according to paired t-test.

^#^ indicates the significance of change over time according to repeated-measures ANOVA.

With regard to parafoveal ChT, ChT immediately (0 min) after complete mydriasis did not significantly decrease at all detected locations except for N_1mm_ (mean change −2±4 μm, P = 0.002). However, the mean changes in parafoveal ChT at 30 and 60 min after thorough pupil dilation showed small but significant decreases (P<0.0001), except for S_3mm_ and I_3mm_ at 30 min (P = 0.363 and P = 0.144, respectively). Similar to the changes in SFChT, ChT at T_1mm_ and N_1mm_ became stable 30 min after complete mydriasis (mean changes: −6±4 μm and −6±4 μm in T_1mm_ and N_1mm_ at 30 min, and −7±5 μm and −7±4 μm in T_1mm_ and N_1mm_ at 60 min, respectively; all P<0.0001). A pairwise comparison of mean changes in parafoveal ChT in T_1mm_ and N_1mm_ showed statistically significant interactions between 0 min and 30 min and between 0 min and 60 min (both P<0.0001), and there was no statistically significant difference between 30 min and 60 min (P = 0.267 for T_1mm_ and P = 0.165 for N_1mm_) (Table 2).

Overall, there was a significant decrease in the SFChT and parafoveal ChT of T_1mm_ and N_1mm_ (all *P<0.0001)_,_ while there was a very small decrease in parafoveal ChT in the other locations according to time after administration of compound tropicamide at 30 and 60 min (Table 2).

### Time-course of changes in other ocular biometrics

The changes in AL, ACD, LT, and K over time after complete mydriasis are shown in Table 3. Overall, AL, LT, and horizontal corneal curvature values became significantly smaller, while ACD became significantly greater, compared with baseline at all time-points (paired t-test; all P<0.05). In addition, there were no interactions between the values and the observation time for all of these parameters (repeated-measures ANOVA, all P>0.05), indicating that the changes happened immediately after complete mydriasis and were maintained within the observation time. In contrast, vertical corneal curvature did not change immediately after complete mydriasis, but then became significantly steeper at 30 min (paired t-test with baseline, P = 0.002) and maintained afterwards (30 min vs 60 min: P>0.05). Nevertheless, it should be pointed out that all of these statistically significant differences should be considered only based on the measurement precision of the Lenstar used in the present study, which is shown in bold in the last two columns in Table 3. This is discussed in detail in the next section.

**Table 3:** Changes in ocular biometry over time after complete mydriasis.

Parameter	Mean change from baseline			ANOVA
	0 min	30 min	60 min	
	Mean±SD (P *)			P ^#^
AL (mm)	−0.01±0.02	−0.01±0.02	−0.02±0.02	0.113
	(0.001)	(0.000)	(0.000)	
ACD (mm)	0.06±0.04	0.07±0.04	0.07±0.04	0.598
	(0.000)	(0.000)	(0.000)	
LT (mm)	−0.03±0.02	−0.03±0.02	−0.03±0.03	0.519
	(0.000)	(0.000)	(0.000)	
K_1_ (D)	−0.05±0.07	−0.05±0.07	−0.06±0.06	0.646
	(0.000)	(0.000)	(0.000)	
K_2_ (D)	−0.03±0.14	−0.07±0.14	−0.07±0.13	0.349
	**(0.144)**	(0.002)	(0.001)	

The time-points were 0, 30, and 60 min after complete mydriasis.

* indicates the significance of the change from baseline according to paired t-test.

^#^ indicates the significance of change over time according to repeated-measures ANOVA. AL: axial length; LT: lens thickness; ACD: anterior chamber depth; K1: horizontal corneal curvature; K2: vertical corneal curvature.

## Discussion

The results of this study demonstrated that compound tropicamide generally decreased ChT, especially at locations directly under the fovea (1 mm temporal and nasal to the fovea). The decreases occurred immediately after complete mydriasis, reached a plateau approximately 30 min later, and were maintained for at least the following 30 min. To the best of our knowledge, this is the first study that has depicted a detailed time-course of the effect of a mydriatic on ChT.

A multitude of studies have investigated the effect of tropicamide or phenylephrine alone or as a mixture on ChT (most referred to SFChT). Since the time-point to measure the ChT varied among the previous studies, it is recalculated as the time after the first instillation of eye drops, in order to facilitate the comparison (Table 4). In agreement with the present study, Kara et al. [25] and Yuvacj et al. [26] both reported decreased SFChT after administration of 1% tropicamide or 2.5% phenylephrine alone. On the other hand, Kim et al. [27] failed to observe significant changes in SFChT with the same agent used in the present study (0.5% tropicamide plus 0.5% phenylephrine). This discrepancy could be due to the significant difference in the participants’ ages and ChTs between the previous study and the present one (33.2±14.3 years vs 11.9±2 years; 313.00±90.05 μm vs 239.44±58.52 μm). We found that, at least in our children, the choroid continued thinning until 70 min after the first instillation; it is therefore essential to compare the results based on the time-points for measurement. Also due to the significant difference, it is nevertheless very difficult to postulate the results if they had extended the observation time.

**Table 4:** Summary of time-points to measure the SFChT across studies.

Study	Eye drop	Time-point of detection	Outcome	
			Mean change of SFChT (μm)	
Kara et al.[25]	1% tropicamide		−22±14	(P<0.001)
	2.5% phenylephrine	55 min	−17±09	(P<0.001)
	Artificial tears		−5.3±3.5	(P = 0.108)
Yuvac et al.[26]	1% tropicamide		−26.55±33.85	(P<0.001)
	2.5% phenylephrine	60 min	−25.72±32.66	(P<0.001)
	1% cyclopentolate		−19.47±19.41	(P<0.001)
Kim et al.[27]	Artificial tears		−9.97±38.98	(P = 0.172)
	Compound tropicamide^#^	40 min	3.34±40.70	(P = 0.500)
	Unpreserved saline		2.07±25.01	(P = 0.248)
The present study	Compound tropicamide^#^	40 min	−5±4	(P<0.001)
		70 min	−12±4	(P<0.001)
		100 min	−13±4	(P<0.001)

To facilitate the comparison between studies, the time-point of detection was recalculated from the time after the first instillation of eye drops for each study. Compound tropicamide^#^: 0.5% tropicamide combined with 0.5% phenylephrine.

The mechanism by which ChT is influenced by mydriatics is not clear but has been proposed in previous studies [25–27]. Briefly, it may include the contraction of nonvascular smooth muscle cells that are innervated by both sympathetic and parasympathetic inputs, the contraction of the choroidal vascular bed due to the vasoconstricting effects of these ingredients, and also the decreased mechanical traction and posterior movement of the ciliary muscle after cycloplegia. However, it should be pointed out that most of the proposed mechanisms are speculative, and as a matter of fact, experiments aimed at determining the exact mechanism have failed to produce consistent results. Thus, more studies are warranted in this regard. Additionally, the mechanism seems to be mydriatic-specific. For instance, atropine and homatropine also demonstrate mydriatic effects, but were found to induce choroidal thickening rather than choroidal thinning [24]. In contrast to tropicamide and phenylephrine, atropine [29, 30] and probably also homatropine [31] have very strong effects in inhibiting myopia. Therefore, they might represent another category of mydriatic that involves an independent mechanism.

Decreases in ChT after treatment with compound tropicamide were accompanied by statistically significant increases in ACD and decreases in LT, K_1_, K_2_, and AL. The changes in ACD and LT in our study were similar to those of previous studies on both animals [32, 33] and humans [24, 34, 35]. This is simply due to the pharmaceutical effects on the pupil-dilatator muscle and ciliary smooth muscle. In the present study, the magnitude of LT thinning was smaller than that of ACD deepening, indicating that the crystal lens and iris plane might move backward due to the pull force of the relaxed ciliary muscle [24, 34–37]. Meanwhile, AL was found to significantly shorten after the administration of the adrenergic and anticholinergic agents used in the study. This finding is in agreement with the observation that sustained accommodation stimulated a transient increase in AL [38, 39]. We concur with the explanation of Mallen et al. that, in contrast to accommodation, application of compound tropicamide resulted in the relaxation of ciliary smooth muscle, applying an outward push-force to a region of the choroid and sclera adjacent to the ciliary body. This effect requires a rearward displacement of the posterior portion of the globe to maintain a constant ocular volume, which leads to a decrease in AL. However, it should be noted that this explanation seems to be paradoxical to the decreased thickness of the choroid observed in the present study. Presumably, ChT should have increased, accompanied with the shrinkage of AL [40], because AL as defined by Lenstar refers to the distance from the corneal epithelium to the RPE at the interior border of the choroid. However, the unexpectedly decreased ChT could be due to the fact that, as mentioned previously, the relaxation of ciliary smooth muscle leads to a pull-force at the posterior portion of the globe, including the sclera, and therefore results in an inward push-force to the exterior border of the choroid by the sclera. When the inward movement of the exterior border of the choroid outweighs the inward movement of the interior border, the ChT could decrease as observed. However, we admit that this theoretical speculation needs to be justified with *in vivo* techniques to measure the actual border of the sclera, which are unavailable at the moment. The definition of AL by Lenstar also sheds some light on another important problem: since AL measured by Lenstar does not take ChT into consideration, the detected magnitude of decrease in AL (20 μm) actually underestimated the true effect of compound tropicamide on AL, which should have been 33 μm in the present study. AL is one of the most essential parameters for evaluating myopic progression, and Lenstar together with another device, IOLMaster, is currently the most widely-used instrument for this measurement. However, both apply the same definition of AL as described above. Thus, it should be kept in mind that ChT has a non-negligible effect on the detected AL, and only those results that consider this important element are validated.

In conclusion, the present study showed that mydriasis with compound tropicamide leads to a decrease in SFChT and parafoveal ChT, but the specific magnitude varies with time. Although these findings should not be directly extrapolated to other mydriatics with other components or concentrations, they highlight the importance of detection time-points for comparing pharmaceutically-induced changes in ChT.

## Supporting Information

S1 Table Data base. (XLSX)
