# Correction: Time-Course of Changes in Choroidal Thickness after Complete Mydriasis Induced by Compound Tropicamide in Children

**DOI:** 10.1371/journal.pone.0171924

**Published:** 2017-02-06

**Authors:** The PLOS ONE Staff

In the Funding section, the grant number from the funder National Natural Science Foundation of China, China is listed incorrectly. The correct grant number is: 81200716. The publisher apologizes for the error.
